# Supplementary material for: A note on the analysis of two-stage task results: How changes in task structure affect what model-free and model-based strategies predict about the effects of reward and transition on the stay probability
Source: PLoS One. 2018 Apr 3;13(4):e0195328. doi: 10.1371/journal.pone.0195328 (PMC5882146; doi:10.1371/journal.pone.0195328)
Supplement: S1 Appendix — (PDF) [file pone.0195328.s001.pdf]

S1 Appendix

We will prove that if $p + b \neq 1$, then there is a transition effect on the results of model-based agents. As explained in the Methods, if each initial-state action transitions to a different final state with the same probability, then the probability $P(\text{left}|s_i)$ of choosing left at the initial state s_i is given by

$$P(\text{left}|s_i) = \frac{1}{1 + \exp[-K(p - b)]} = \text{logit}^{-1} K(p - b), \tag{17}$$

where $K \geq 0$ is a constant that depends on the transition probabilities and the exploration-exploitation parameter.

According to the model-based reinforcement learning rule (Equation 14), if the agent chooses left, then experiences a common transition to pink and receives 1 reward, the stay probability p_{stay} (of choosing left again in the next trial) is given by

$$p_{\text{stay}} = \text{logit}^{-1} K[(1 - \alpha)p + \alpha - b]; \tag{18}$$

if instead the agent experiences a rare transition to blue and receives 1 reward, p_{stay} is given by

$$p_{\text{stay}} = \text{logit}^{-1} K[p - (1 - \alpha)b - \alpha]; \tag{19}$$

if the agent experiences a common transition to pink and receives 0 rewards, p_{stay} is given by

$$p_{\text{stay}} = \text{logit}^{-1} K[(1 - \alpha)p - b]; \tag{20}$$

and if the agent experiences a rare transition to blue and receives 0 rewards, p_{stay} is given by

$$p_{\text{stay}} = \text{logit}^{-1} K[p - (1 - \alpha)b]. \tag{21}$$

The logistic regression model, on the other hand, determines p_{stay} as a function x_r ($x_r = +1$ for 1 reward, $x_r = -1$ for 0 rewards in the previous trial) and x_t ($x_t = +1$ for a common transition, $x = -1$ for a rare transition in the previous trial):

$$p_{\text{stay}} = \text{logit}^{-1}(\beta_0 + \beta_r x_r + \beta_t x_t + \beta_{r\times t} x_r x_t). \tag{22}$$

Since logit^{-1} is a one-to-one function, this implies that

$$K[(1-\alpha)p+\alpha-b] = \beta_0+\beta_r+\beta_t+\beta_{r\times t}, \tag{23}$$

$$K[p-(1-\alpha)b-\alpha] = \beta_0+\beta_r-\beta_t-\beta_{r\times t}, \tag{24}$$

$$K[(1-\alpha)p-b] = \beta_0-\beta_r+\beta_t-\beta_{r\times t}, \tag{25}$$

$$K[p-(1-\alpha)b] = \beta_0-\beta_r-\beta_t+\beta_{r\times t}. \tag{26}$$

Solving this system for β_0, β_r, β_t, and $\beta_{r\times t}$ yields

$$\beta_0 = K\left(1-\frac{\alpha}{2}\right)(p-b), \tag{27}$$

$$\beta_r = 0, \tag{28}$$

$$\beta_t = K\frac{\alpha}{2}(1-p-b), \tag{29}$$

$$\beta_{r\times t} = K\frac{\alpha}{2}, \tag{30}$$

which implies that if $\alpha > 0$, $K > 0$ and $p+b \neq 1$, then $\beta_t \neq 0$. This proof assumes that the agent chose left, but the same can be proved if the agent chose right, as in this example "left," "right," "pink," and "blue" are arbitrary.
